# Ecdysone and 20-hydroxyecdysone are not required to activate glycolytic gene expression in *Drosophila melanogaster* embryos

**DOI:** 10.17912/micropub.biology.000501

**Published:** 2021-11-30

**Authors:** Jason M. Tennessen

**Affiliations:** 1 Indiana University

## Abstract

Many of the *Drosophila *enzymes involved in carbohydrate metabolism are coordinately up-regulated approximately midway through embryogenesis. Previous studies have demonstrated that this metabolic transition is controlled by the *Drosophila *Estrogen-Related Receptor (dERR), which is stabilized and activated immediately prior to onset of glycolytic gene expression. The mechanisms that promote dERR activity, however, are poorly understood and other transcriptional regulators could control this metabolic transition, independent of dERR. In this regard, the steroid hormone 20-hydroxyecdysone (20E) represents an intriguing candidate for regulating glycolytic gene expression in embryos – not only does the embryonic 20E pulse immediately precede transcriptional up-regulation of glycolytic metabolism, but 20E is also known to promote *Lactate dehydrogenase *gene expression. Here I test the hypothesis that embryonic 20E signaling is required to activate glycolytic gene expression. Using developmental northern blots, I demonstrate that the transcriptional up-regulation of glycolytic genes during embryogenesis still occurs in *shadow *mutants, which are unable to synthesize either ecdysone or 20E. My finding indicates that ecdysone and 20E signaling are not required for this mid-embryonic metabolic transition.

**Figure 1. Timecourse northern blots examining glycolytic gene expression in control and shadow mutant embryos:**
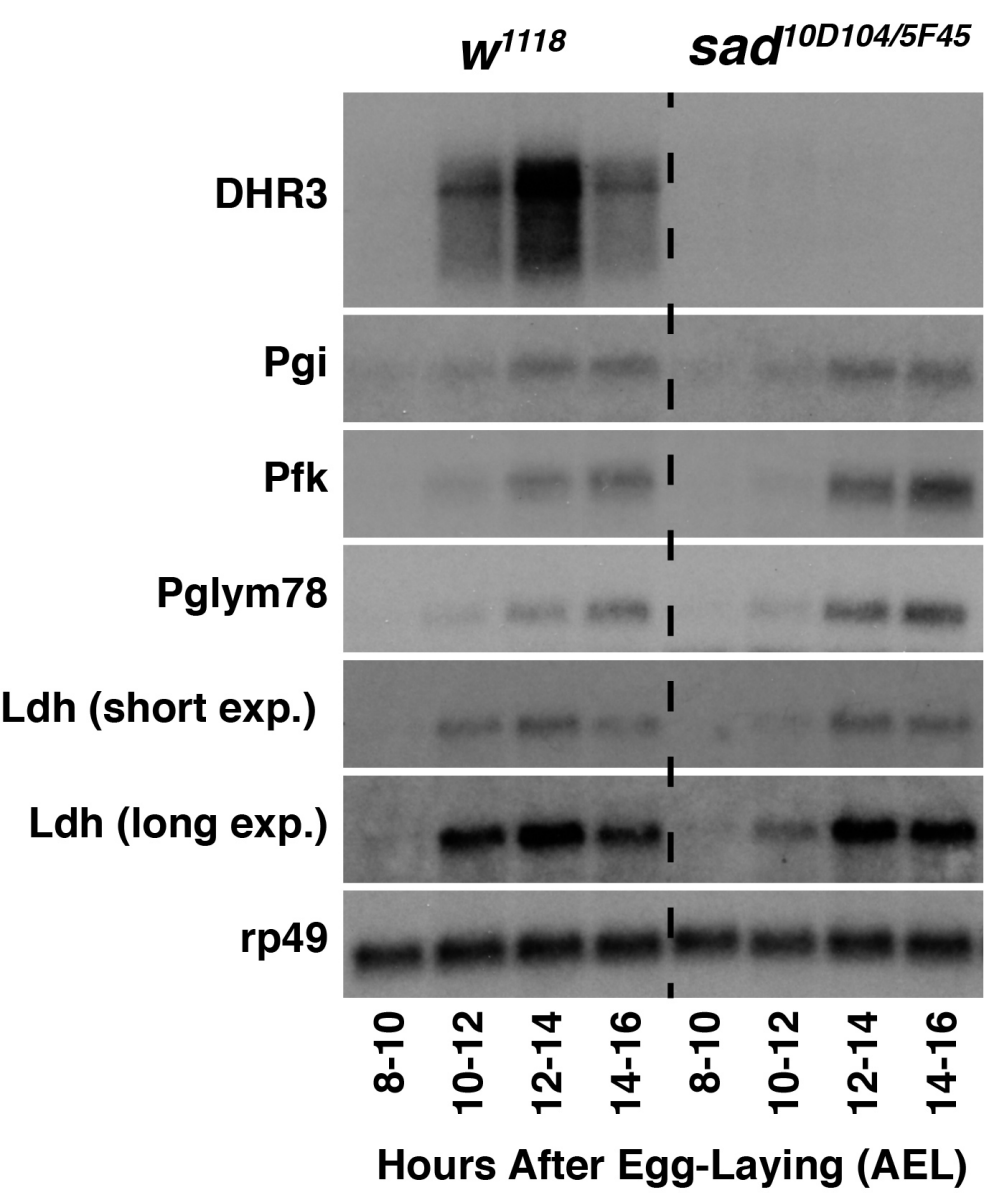
Total RNA from stage *w^1118^* control embryos and and *w^1118^; shadow^10D104/5F45 ^*mutantembryos were analyzed by northern blot hybridization to detect transcripts encoding DHR3, Pgi, Pfk, Pglym78, and Ldh. The Ldh short exposure represents a 6-hour long film exposure. The Ldh long exposure is the same blot exposed for 24 hours. Hybridization to detect *rp49* mRNA is included as a loading control.

## Description

During the course of the *Drosophila* development, metabolism readily adapts to meet the energetic and biosynthetic demands of each life stage (Gillette *et al.*, 2021). This relationship between growth and metabolism is particularly apparent when examining changes in glycolytic gene expression. Approximately midway through embryonic development, transcripts representing nearly every enzyme involved in glycolysis are coordinately up-regulated (Abu-Shumays and Fristrom, 1997; Currie and Sullivan, 1994a, b; Madhavan *et al.*, 1972; Roselli-Rehfuss *et al.*, 1992; Shaw-Lee *et al.*, 1992; Shaw-Lee *et al.*, 1991; Sun *et al.*, 1988; Tennessen *et al.*, 2011; Tennessen *et al.*, 2014; Tixier *et al.*, 2013; Wright and Shaw, 1970). The resulting glycolytic program is maintained throughout the larval growth period and subsequently down-regulated prior to the onset of metamorphosis (White *et al.*, 1999). These metabolic transitions are highly predictable and provide an opportunity to understand how metabolism adapts to meet the energetic demands of insect development.

Activation of glycolytic metabolism in *Drosophila* embryos depends on the *Drosophila* Estrogen-Related Receptor (dERR; FBgn0035849), which represents the sole fly ortholog of the orphan class of ERR nuclear receptors (Ostberg *et al.*, 2003). In embryos cultured at 25ºC, dERR becomes transcriptionally active ~10-12 hours after oviposition, resulting in the coordinate up-regulation of genes involved in carbohydrate metabolism (Tennessen *et al.*, 2011). The mechanisms that temporally regulate dERR activity during this metabolic switch, however, remain unknown and additional factors could be controlling embryonic glycolytic metabolism independent of dERR. In this regard, both dERR activation and onset of glycolytic gene expression correlate with the embryonic pulse of the steroid hormone 20-hydroxyecdysone (20E), which triggers dorsal closure, cuticle deposition, head involution, and a variety of other developmental events (Chavez *et al.*, 2000; Kozlova and Thummel, 2003; Maróy *et al.*, 1988; Warren *et al.*, 2002). Moreover, not only is 20E known to induce *Lactate Dehydrogenase* (*Ldh*; FBgn0001258)expression in imaginal discs (Abu-Shumays and Fristrom, 1997), but the *Ecdysone Receptor* (*EcR*; FBgn0000546) and dERR are also reported to cooperatively regulate expression of glycolytic genes in insect cell culture (Kovalenko *et al.*, 2019), thus raising the possibility that 20E-signaling is necessary for the up-regulation of embryonic carbohydrate metabolism.

To test the hypothesis that 20E transcriptionally regulates glycolysis in *Drosophila* embryos, I used developmental northern blots to analyze expression of key glycolytic genes in *shadow* (*sad*; FBgn0003312) mutants, which are unable to synthesize either ecdysone or 20E (Warren *et al.*, 2002). As a positive control, I first analyzed expression of *DHR3* (FBgn0000448), which is up-regulated in response to embryonic 20E production (Ruaud *et al.*, 2010). Unlike *w^1118^* control samples that expressed *DHR3* during mid-embryogenesis, *DHR3* transcripts were undetectable in *sad* mutant samples – a result consistent with lack of 20E production in these embryos. In contrast, *sad* mutants still exhibit the coordinate up-regulation of *Phosphoglucoisomerase* (*Pgi*; FBgn0003074), *Phosphofructokinase* (*Pfk*; FBgn0003071), and *Phosphoglyceromutase 78* (*Pglym78*; FBgn0014869) transcripts during mid-embryogenesis, indicating that 20E is not necessary to activate glycolytic gene expression during mid-embryogenesis. I would note, however, that *Ldh* transcripts levels were noticeably decreased at the 10-12 hour AEL timepoint in *sad* mutant samples when compared with controls, suggesting that 20E partially regulates *Ldh* expression at this early timepoint. Such a possibility is consistent with previous observations that 20E activates *Ldh* expression in imaginal discs and that embryonic Ldh mRNA transcript levels are elevated at an earlier timepoint when compared with other glycolytic genes (Abu-Shumays and Fristrom, 1997; Tennessen *et al.*, 2011).

Overall, my findings suggest that that 20E signaling is not required for the coordinate up-regulation of glycolytic gene expression observed midway through embryonic development. All glycolytic genes examined in this study exhibited normal up-regulation in *sad* mutants, with the only exception being *Ldh*, which exhibited slightly delayed onset of expression when compared with the control strain. My finding, however, does not exclude the possibility that ecdysone or 20E controls glycolytic metabolism within individual cells or tissues that would be overlooked using northern blot analysis. Finally, my study further highlights the role of dERR as a central regulator of carbohydrate metabolism during mid-embryogenesis and again raises the question as to what developmental signals induce dERR activity during this metabolic transition.

## Methods

*Drosophila *Genetics and Embryo Collection: All strains were maintained on Bloomington *Drosophila* Stock Center (BDSC) media. Strains containing the *sad alleles sad^10D104^* and *sad^5F45^* (a kind gift from Dr. Michael O’Connor) were individually crossed to BDSC stock #6663 (*w^1118^; Dr^Mio^/TM3, P{w[+mC]=GAL4-twi.G}2.3, P{UAS-2xEGFP}AH2.3, Sb^1^ Ser^1^*). F1 male offspring were again crossed into BDSC strain #6663 and progeny lacking the *Dr^Mio^* chromosome were used to establish balanced stocks.

Egg collection and synchronization were conducted as previously described (Li and Tennessen, 2017). For both *w^1118^* controls and *sad* mutants, 50 females and 25 males were placed in an egg-laying bottle with a molasses agar plate containing a smear of yeast paste taped in the lid. In the case of *sad* mutant embryo collections, *sad^10D104^/TM3, P{w[+mC]=GAL4-twi.G}2.3, P{UAS-2xEGFP}AH2.3, Sb^1^ Ser^1^* virgin females were crossed with *sad^5F45^/TM3, P{w[+mC]=GAL4-twi.G}2.3, P{UAS-2xEGFP}AH2.3, Sb^1^ Ser^1^* males. *sad^10D104/5F45^* mutant embryos were identified by the absence of GFP expression using a Zeiss SterREO Discovery V8 microscope.

Northern Blots: RNA preparation and northern blot analysis was conducted using a previously described method (Karim and Thummel, 1992). Briefly, staged embryos were dechorionated and RNA extracted using Trizol Reagent (Life Technologies) following the manufacturers protocol. 3 µg total RNA from each sample was individually added to a 1.5 ml microcentrifuge tube containing a premixed solution of 3 µl 10X Formaldehyde Gel Buffer (0.2 M MOPS pH 7.0, 50 mM Sodium Acetate, 10 mM EDTA), 3.5 µl of formaldehyde (37% w/v), and 10 µl of formamide. Sample volume was adjusted to 25 µl by adding the appropriate volume of nuclease free H_2_O, incubated at 65ºC for 5 minutes, and centrifuged briefly at 10,000 x g. 3 µl of loading dye (80% glycerol, 1 mM EDTA, 0.4% bromophenol blue, 0.4% xylene cyanol) was added to the sample and mixed by pipetting. The entire sample was then loaded into a formaldehyde-agarose gel (1 g agarose, 75 mL H_2_O, 10 mL of 10X formaldehyde gel buffer, 15 mL of 37% formaldehyde [m/v]; immersed within 1X formaldehyde gel buffer). RNA samples were separated at 70 volts for 150 minutes.

RNA was transferred overnight from the gel to GeneScreen Hybridization Transfer Membrane (Perkin Elmer) using standard blotting techniques and crosslinked to the membrane using the “Autocrosslink” setting on a Stratagen UV Stratalinker 1800. Prior to hybridization, membranes were pretreated for one hour at 42ºC with 10 ml of hybridization buffer (5 ml of formamide, 2 ml of 10X PIPES buffer [0.1 M PIPES, pH6.5, 4 M NaCl], 1 ml of 10% SDS, 2 ml H_2_0, 100 µl sheared herring sperm DNA).

For Pfk, Pgi, Pgylm78, and Ldh, radioactive probes were generated from PCR amplified cDNA fragments (see reagents table for oligos used to synthesize cDNA fragments). For DHR3 and rp49, probes were generated as previously described (Sullivan and Thummel, 2003). Labeling reactions were conducted with ^32^P-labeled dCTP ([a-32P]- 3000Ci/mmol 10mCi/ml EasyTide, 250 µC; Perkin Elmer; BLU513H250UC) using a Prime-It II Random Primer Labeling Kit (Agilent Catalog #300385) following the manufacturer’s instructions. Individual reactions were cleaned using a MicroSpin G-50 Sephadex column (Amersham Catalog #27533001). Radioactive probes were added to 100 µl of sheared herring sperm DNA in a 2 ml screwcap tube, boiled for 5 minutes, and added to the hybridization tube containing the membrane and buffer.

Labeled probes were allowed to hybridize with the membrane overnight at 42ºC, at which time the hybridization buffer/probe mixture was poured out of the hybridization tube and the membrane washed twice for 10 minutes at 42ºC with 10 ml of 2X SSC + 0.1% SDS, once for 10 min at 55ºC with 10 ml of 1X SSC + 0.1% SDS, and once for 10 min at 55ºC with 10 ml of 0.1X SSC + 0.1% SDS. After the final wash, the membrane was removed from the hybridization tube, briefly immersed in 2X SSC, wrapped in plastic wrap, sandwiched between two intensifying screens with a piece of film, and placed at -80ºC for 24 hours. Exposed film was developed using a Kodak X-OMAT film processor.

## Reagents

*Oligo sequences used to generate northern blot probes*

**Table:** 

**Gene**	**Oligo sequences**
Pfk	5’- ATGCATTCAATAAAATTTCGAGTATTTACC-3’ 5’- TTAGGCGACGGCGTCAGTGTCAC-3’
Pgi	5’- ATGGCCGGCCCACTTCCTCC -3’ 5’- TTACTTCCAATTGGCTTTGATG-3’
Pglym78	5’- CCACTACGGTGGACTCACTG -3’ 5’- ATGGCCTTCTTCACGGTCTC -3’
Ldh	5’- ATGGCCGCCATTAAGGACAGTCTG -3’ 5’- TTAGAACTTCAGACCAGCCTGGAC -3’

**Table:** 

**Strain**	**Genotype**	**Available from**
5905	*w[1118]*	BDSC
JMT49	*w[1118]; sad[10D104], st, e/TM3, P{w[+mC]=GAL4-twi.G}2.3, P{UAS-2xEGFP}AH2.3, Sb[1] Ser[1]*	Tennessen Lab
JMT54	*w[1118]; sad [5F45] ru, h, th, st, cu, sr, e/TM3, P{w[+mC]=GAL4-twi.G}2.3, P{UAS-2xEGFP}AH2.3, Sb[1] Ser[1]*	Tennessen Lab
